# A machine learning analysis of suicidal ideation and suicide attempt among U.S. youth and young adults from multilevel, longitudinal survey data

**DOI:** 10.3389/fpsyt.2025.1511966

**Published:** 2025-02-24

**Authors:** Molly M. Jacobs, Anne V. Kirby, Jessica M. Kramer, Nicole M. Marlow

**Affiliations:** ^1^ Department of Health Services Research, Management and Policy, College of Public Health and Health Professions, University of Florida, Gainesville, FL, United States; ^2^ Department of Occupational and Recreational Therapies, College of Health, University of Utah, Salt Lake City, UT, United States; ^3^ Department of Occupational Therapy, College of Public Health and Health Professions, University of Florida, Gainesville, FL, United States

**Keywords:** suicide attempt, suicidal ideation, adolescents and young adults, socioecological framework, machine learning, longitudinal data

## Abstract

**Objectives:**

To investigate individual, interpersonal, health system, and community factors associated with suicidal ideation (SI) and attempts (SA).

**Methods:**

Utilizing nationally representative data from the National Longitudinal Study of Adolescent to Adult Health (7^th^-12^th^ graders in 1994-95 followed >20 years until 2016-18, N=18,375), least absolute shrinkage selector operator (LASSO) regression determined multilevel predictors of SA and SI. Models comprised full and diagnosis subgroups (ADD/ADHD, depression, PTSD, anxiety, learning disabilities [LD]).

**Results:**

Approximately 2.48% and 8.97% reported SA and SI, respectively. Over 25% had depression, and 20.98% anxiety, 6.42% PTSD, 4.55% ADD/ADHD, and 2.50% LD. LASSO regression identified 20 and 21 factors associated with SA and SI. Individual-level factors associated with SI and SA included educational attainment, substance use, ADD/ADHD, depression, anxiety, and PTSD. Interpersonal-level factors included social support, household size, and parental education, while health system-level factors comprised health care receipt, health insurance, and counseling. The strongest associations were among individual-level factors followed by interpersonal and health system factors.

**Conclusions:**

The distinct factors associated with SI and SA across diagnostic subgroups highlight the importance of targeted, subgroup-specific suicide prevention interventions. These findings emphasize the value of precise, data-driven approaches for suicide prevention among diverse populations and individuals with disabilities across the life-course.

## Introduction

Suicide is a major public health concern in the U.S., accounting for nearly 50,000 deaths in 2022 (14.3/100,000), the highest rate since 1941 (1). Suicidal thoughts and behaviors (STB), including suicidal ideation (SI) and suicide attempts (SA), affected >3M youth and >13M adults in 2022 (2). Despite significant progress, U.S. suicide rates continue to rise and disparities in STB exist (3). For example, evidence shows that individuals with disabilities such as attention deficit disorder/attention deficit hyperactivity disorder (ADD/ADHD), depression, anxiety, post-traumatic stress disorder (PTSD), and learning disabilities (LD) have higher risk (4–8). To address these issues, the 2024 *National Strategy for Suicide Prevention* (*National Strategy*) calls for coordinated and comprehensive public health approaches to suicide prevention, reflecting a multilevel perspective (3).

Successful implementation of comprehensive prevention approaches will require innovative data and research (3), such as multilevel, socioecological examinations of mutable risk factors most closely associated with STB (3, 9). Socioecological theories indicate multilevel factors that influence suicide risk, including individual (e.g., age, sex, race/ethnicity, LGBTQIA+ identity, mental illness, social isolation), interpersonal/relationship (e.g., loss of relationship(s), high conflict or violent relationships, social exclusion), community (e.g., community violence, lack of access to health care or other services), and societal/policy factors (e.g., access to lethal means of suicide, U.S. region, economic downturn, mental health funding) (3, 9).

A multilevel, socioecological framework (9) is critical for developing community resources and health policies that target social determinants of health (SDOH)—the non-medical factors that influence health outcomes including the conditions in which people are born, grow, live, work, and age—in populations disproportionately affected by STB (3), including people with disabilities, across the life-course. Socioecological frameworks synthesize the relationships and interactions between social and ecological factors by conceptualizing how multiple, interconnected levels of context influence individual behaviors and outcomes. These frameworks typically organize influences into nested layers, such as individual, interpersonal, community, organizational, and societal or policy levels. They emphasize the dynamic interplay between people and their environments, recognizing that individual choices and health outcomes are shaped by broader social determinants, such as cultural norms, socioeconomic status, and community infrastructure, as well as ecological conditions, such as climate, natural resources, and geographic factors. By integrating these layers, socioecological frameworks provide a comprehensive approach to understanding complex phenomena and designing interventions that address systemic interactions across multiple levels of influence.

SDOH, including economic instability, education, neighborhood environment, healthcare access, social support, and discrimination, significantly influence the risk of SA and SI. Economic insecurity, such as poverty and unemployment, heightens stress and limits access to resources, increasing suicide risk (10). Limited educational attainment is associated with poorer mental health outcomes due to reduced opportunities and lower health literacy (11). Unsafe living conditions and exposure to violence in deprived neighborhoods further exacerbate vulnerability (12). Disparities in access to mental health services, often driven by geographic or financial barriers, leave many individuals without adequate care (13). Social isolation and lack of support are key risk factors, as strong social connections are protective against STB (14). Additionally, discrimination and stigma faced by marginalized populations, including racial and ethnic minorities and LGBTQ+ individuals, amplify mental health disparities and suicide risks, as highlighted by minority stress theory (15). Addressing these interconnected determinants is essential for effective suicide prevention strategies.

While studies have identified several multilevel factors associated with SI and SA in various racial, geographic, and social cohorts (9), few studies have examined socioecological characteristics associated with STB in nationally representative samples. To expand our understanding of multilevel factors and inform development of resources and policies that address SDOH within suicide prevention efforts, this study used the National Longitudinal Study of Adolescent to Adult Health (Add Health) to identify distinct sets of socioecological characteristics associated with SA and SI among youth and young adults and whether differential patterns would emerge between subgroups of individuals with known high risk conditions assessed in Add Health, including ADD/ADHD, depression, anxiety, PTSD, and LD (4–8).

## Materials and methods

### Data

Data were utilized from Add Health—a nationally representative, longitudinal survey of adolescents (grades 7–12) during the 1994–1995 school year in the U.S. Add Health includes longitudinal data on respondents’ social, economic, psychological and physical well-being with contextual data, providing unique opportunities to study how health, social environments, and behaviors are linked over time. The cohort was followed into young adulthood with five in-home interviews during 1995 (Wave I, N=20,745), 1996 (Wave II, N=17,738), 2001–02 (Wave III, N=15,197), 2008–09 (Wave IV, N=15,701), and 2016–18 (Wave V, N=12,300) when respondents were 12-17, 13–18, 18–26, 24–32, and 33-43 years old, respectively. For additional information on Add Health, see http://www.cpc.unc.edu/projects/addhealth/design. Data was acquired through the Add Health Restricted-Use Data Contract #19101801. The study was reviewed and approved by the University of Florida institutional review board (#IRB202102130).

### Outcome variables

In each Wave, respondents were asked if they had seriously thought about committing suicide in the past 12 months (SI). Those who responded affirmatively were then asked how many times they attempted suicide (SA) as a follow-up question. However, due to inconsistency in the answer choices presented in each wave, we could not consistently enumerate the number of attempts between waves. Therefore, SA responses were coded as zero (no attempts) or one (at least one attempt).

### Candidate variables

Candidate variables included potential predictors of or explanatory factors related to suicide, but excluded variables that were themselves outcomes of SI or SA [such as non-suicidal self-injury (NSSI) or other self-harm behaviors that may occur after a suicide attempt], or otherwise predetermined since their inclusion would bias the focal association (16). Therefore, to capture multilevel socioecological factors (3, 9), sets of theoretically relevant individual, interpersonal, health system, and community characteristics were selected. **Table 1** presents the survey wave of collection for each item indicating the applicable time frame for all factors.

**Table 1:** Factors by wave.

Factor	Wave I	Wave II	Wave III	Wave IV	Wave V	Parental Survey (Wave I, IV)
Age	x	X	x	x	x	
Sex	x	x	x	x	x	
Race	x	x	x	x	x	
Ethnicity	x	x	x	x	x	
Same-sex romantic attraction	x	x	x	x	x	
Self-reported health	x	x	x	x	x	
BMI	x	x	x	x	x	
Highest education	x	x	x	x	x	
Health insurance	x	x	x	x	x	
Received needed healthcare	x	x	x	x	x	
Mental health counseling	x	x	x	x	x	
Smoking	x	x	x	x	x	
Alcohol	x	x	x	x	x	
Marijuana	x	x	x	x	x	
Regular exercise	x	x	x	x	x	
TV hours	x	x	x	x	x	
Age at intercourse	x	x	x	x	x	
Working	x	x	x	x	x	
In school	x	x	x	x	x	
Household size	x	x	x	x	x	
Social support	x	x	x	x	x	
Region	x	x	x	x	x	
Distance HOLC	x	x	x	x	x	
Parent’s education						x
Parent’s married						x
Walker/crutches						x
Special educational accommodations						x
LD	if reported in any wave					
ADHD	if reported in any wave					
Depression	if reported in any wave					
Anxiety	if reported in any wave					
PTSD	if reported in any wave					

### Individual

Individual factors included demographic (age, sex, race, ethnicity, highest educational attainment, same sex romantic attraction), health/disability (self-reported health status, body mass index [BMI], using an assistive device such as a walker or crutches, and self- or parent-reported diagnosis of LD, ADD/ADHD, depression, anxiety, and PTSD), and behavioral (smoking, alcohol consumption, marijuana use, regular exercise, hours of television viewing, employment [≥10 hours/week], school-enrollment [at least part-time when surveyed], sexual activity, age of first sexual intercourse) characteristics.

### Household/family (interpersonal)

Household/family characteristics included number of household residents, youth’s perceived social support, parent’s highest educational attainment, household income level, and parent’s marital status.

### Health systems

Service systems factors included having health insurance, having received needed health services, having received/receiving special educational accommodations in school, and having received mental health counseling within the past 12 months.

### Community

Only two societal factor indicators were available, related to locality: region of residence (Northeast, Midwest, South, West) and distance from a historically “redlined” neighborhood. “Redlining” was a systemic practice implemented by the federal government and financial institutions in the mid-20^th^ century wherein neighborhoods were color-coded on maps by the Home Owners’ Loan Corporation (HOLC) to indicate their perceived investment risk. Areas predominantly inhabited by Black, immigrant, and low-income residents were typically outlined in red and classified as “hazardous” for investment. Since the legacy of redlining continues to influence contemporary issues, including housing segregation, educational disparities, healthcare access, and environmental injustices, these neighborhoods are more likely to experience concentrated poverty and adverse health outcomes (17, 18). Distance from these “red” neighborhoods was categorized as <4.99 miles and ≥5 miles.

### Analysis

Initially, we used chi-square tests with 95% confidence intervals to assess associations between study characteristics and SI and SA. We removed variables with >50% missing values from the analysis (19). Missing values of <50% were imputed using multivariate imputation by chained equations (MICE) method (20). Due to potential multicollinearity, neither multivariable regression nor conventional methods of variable selection were suitable because longitudinal observations on the same individual tend to be intercorrelated (21).

Regularization is designed to generalize models with highly complex relationships by adding a penalty to model parameters, so the model generalizes the data instead of overfitting. Least Absolute Shrinkage Selector Operator (LASSO) is a regularization technique that minimizes overfitting by applying a penalty term (λ) to the log-likelihood function, setting coefficients that contributed most to the error to zero. LASSO (22) has been used in a variety of settings with similar sets of variables for outcomes with complex underlying factors (23–25). Hence this technique is useful for analyzing large datasets with demographic, housing statistic, and economic variable indicators.

The general LASSO penalized regression assumes that error terms are independent and have equal variance across observations, which is not the case in longitudinal data. Therefore, we applied the Longitudinal Graphical LASSO (26) which first maximizes the penalized likelihood function to generate a sparse network, representing the precision matrix, then computes the maximum likelihood estimates of the precision matrix and correlation parameters for the given network structure.

Longitudinal graphical LASSO identifies and estimates time-varying relationships between variables in longitudinal data, incorporating sparsity to make complex dependency structures interpretable. It uses graphical models to represent variables as nodes and conditional dependencies as edges. The results reveal how these relationships evolve over time, highlighting dynamic interactions and changes in network structures. This method is particularly useful for understanding dynamic systems, such as evolving social or biological networks, by uncovering when and how specific factors interact over time. However, it does not establish causality and is sensitive to parameter selection, emphasizing the need for careful interpretation and validation of the results.

**Figure 1:**
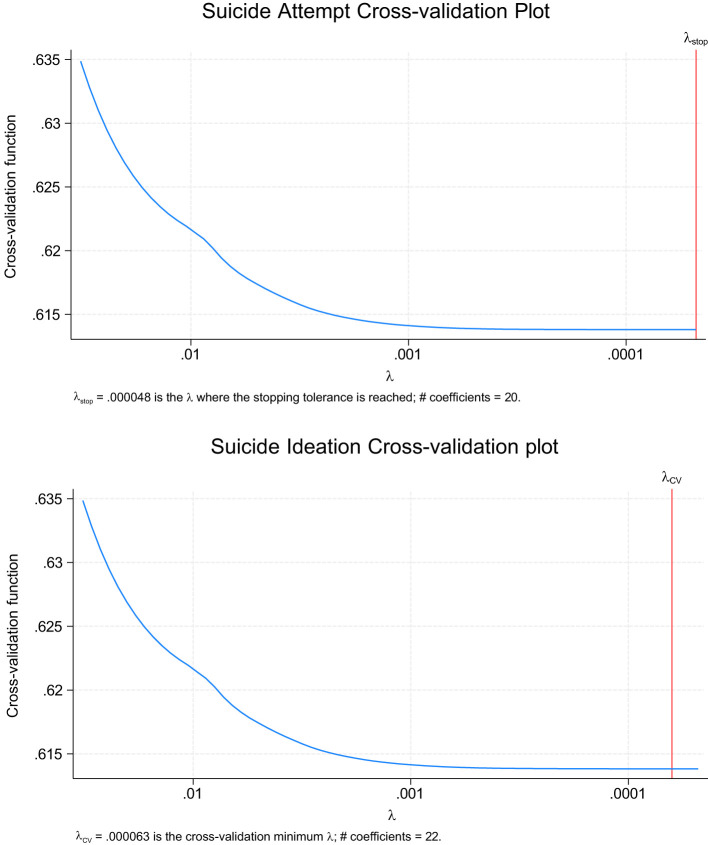
Suicide ideation and attempt cross-validation plots for the full sample.

**Figure 1** confirms that the LASSO method was appropriate compared to other techniques. We fit the LASSO regression models using the “lglasso” package (27) in R software (28), applied separately for SI and SA. We selected a random training set (70%) to train the modes and random hold-out test set (30%) to assess its performance. The training set was used to build the model, and the test set examined the performances of the models. To ensure model results were not influenced by multicollinearity, variance inflation factors (VIF) were inspected. All VIFs were below five, suggesting a low correlation with other factors.

We used 10 × 10-fold cross validation on the training set for our model development to avoid overfitting and to increase generalizability of the machine learning (ML) model. Ten-fold cross-validation was used to select the largest λ within one standard error of the minimum binomial deviance. Performance of the ten models were averaged to create a single performance estimate for that model, and this procedure was repeated 10 times.

To interpret results from the LASSO regression model, the magnitude of the coefficients was used to determine the strength of association between each feature and target variable. Features with larger magnitude coefficients were considered stronger or more important. We also considered the direction of the coefficient when we evaluated the association between each feature and target variable (29). A positive coefficient indicates a positive association, while a negative coefficient indicates a negative association. Features with coefficients close to zero could be considered as having little or no association. To evaluate model performance, model prediction was tested using the testing dataset. Confusion matrix, F1 score, Area Under the Curve (AUC), accuracy, precision, and recall were calculated. AUC provides a standard metric to compare the performance of different models, including those fitted using lasso regression, other types of regularization (like ridge or elastic net), or even non-linear models. By comparing AUC values, you can assess whether lasso regression effectively balances feature selection and predictive performance. The AUC evaluates whether the selected features and the fitted model provide strong predictive accuracy for the classification problem.

To further understand factors associated with STB, we conducted subgroup analyses for specific diagnosis groups. Some studies (4–7) have suggested that individuals with ADD/ADHD, depression, PTSD, and LD have a higher risk of STB than individuals without these diagnoses due to differential experiences in their homes, communities, and society. However, other studies present contradictory findings. To address these inconsistencies, LASSO regression was analyzed among each diagnosis subgroup to evaluate for observed differences.

## Results

### Sample characteristics

Across Waves I through V, respectively, the data contained 806 (3.89%), 507 (3.44%), 235 (1.55%), 221 (1.41%), and 117 (1.47%) respondents with at least one SA and 2,748 (13.25%), 1,570 (10.65%), 891 (5.87%), 1,041 (6.63%), and 784 (6.52%) respondents who reported SI. There were 20,743 (**Table 2**) unique individuals in our sample; 49.47% were males and 50.52% were females. Over 25% had been diagnosed with depression, while 20.98%, 6.42%, 4.55%, and 10.96% reported an anxiety disorder, PTSD, ADD/ADHD, and LD, respectively. On average, respondents were 15.65 (SD=1.75), 16.24 (SD=1.64), 21.98 (SD=1.78), 28.52 (SD=1.77), and 37.56 (SD=1.89) years old in Waves I, II, III, IV, and V. Most were White (61.45%), while 23.17% and 16.99% reported being Black and Hispanic, respectively. While 4.18% had not completed high school or received a GED, 55.09% had completed at least high school or post-high school training (e.g., associate/vocational degree), and 40.72% had a 4-year college degree or higher.

**Table 2:** Sample descriptive characteristics: add health respondents.

	N	Percent			
Male	10263	49.47			
Female	10480	50.52			
Hispanic	3525	16.99			
White	12411	61.45			
Black	4807	23.17			
Anxiety Diagnosis	4352	20.98			
Depression Diagnosis	5229	25.21			
Learning Disability Diagnosis	2276	10.96			
PTSD Diagnosis	1096	6.42			
ADHD	812	4.55			
Education < high school	867	4.18			
Education high school/associate/vocational	11428	55.09			
Education college or above	8448	40.72			
Mean (Percent)	Wave I	Wave II	Wave III	Wave IV	Wave V
Attempt	806 (3.89%)	507 (3.44%)	235 (1.55%)	221 (1.41%)	117 (1.47%)
Ideation	2,748 (13.25%)	1,570 (10.65%)	891 (5.87%)	1,041 (6.63%)	784 (6.52%)
Age	15.65 (SD=1.75)	16.24 (SD=1.64)	21.98 (SD=1.78)	28.52 (SD=1.77)	37.56 (SD=1.89)
Household Size	4.31 (SD=1.16)	4.30 (SD=1.18)	2.64 (SD=1.55)	2.55 (SD=1.50)	2.04 (SD=1.57)

### LASSO regression

The LASSO models were applied to the full sample and five diagnostic subgroups—ADD/ADHD, PTSD, Anxiety, Depression, and LD—to identify multilevel factors significantly associated with SI and SA. All LASSO models followed a similar specification. **Table 2** provides the model fit diagnostics, model accuracy, and LASSO coefficients for the full sample and subgroup SI and SA regressions. As previously indicated, the LASSO coefficients indicate the strength of associations with larger coefficients considered more important factors. Therefore, factors in **Table 3** are ordered by their magnitude. Below the list of features, the total number of identified factors at each level were calculated. **Figure 1** shows the cross-validation convergence for SI and SA for the full sample model. Model-specific results are discussed below.

**Table 3:** LASSO regression results.

Full Sample				ADHD Diagnosis			
Attempt		Ideation		Attempt		Ideation	
Factors	20		21	Factors	4		15
BD	0.610		0.798	BD	0.660		0.846
MCE	0.095		0.141	MCE	0.104		0.153
AUC	0.601		0.600	AUC	0.565		0.584
MSE	0.170		0.239	MSE	0.185		0.257
MAE	0.400		0.479	MAE	0.371		0.515
ACC	0.905		0.859	ACC	0.896		0.867
Intercept	-3.562	Intercept	-2.450	Intercept	-2.801	Intercept	-1.960
Education: < High School	1.396	Education: < High School	0.497	Education: < High School	0.671	Received Counseling	0.242
Received Counseling	0.217	Received Counseling	0.292	Received Counseling	0.113	Depression Diagnosis	0.147
Education: High School	-0.096	Depression Diagnosis	0.182	Depression Diagnosis	0.011	Education: < High School	0.143
Depression Diagnosis	0.091	Not Received Needed Care	0.139	Age	0.000	Same Sex Romantic Attraction	0.137
Not Received Needed Care	0.082	Same Sex Romantic Attraction	0.128			Alcohol Consumption	0.086
Same Sex Romantic Attraction	0.055	Alcohol Consumption	0.109	IND	3	Not Received Needed Care	0.056
Alcohol Consumption	0.049	Regular Smoking	0.090	FAM	0	Learning Disability Diagnosis	-0.044
Uses Marijuana	0.042	Uses Marijuana	0.077	HS	1	Anxiety Diagnosis	0.041
Anxiety Diagnosis	0.040	Education: High School	-0.058	COM	0	Regular Smoking	0.035
Regular Smoking	0.033	Anxiety Diagnosis	0.057			Social Support	-0.012
Parent Education: < High School	0.027	Enrolled in School	0.051			Poor Health	0.011
PTSD Diagnosis	0.023	Poor Health	0.042			Female	0.009
Enrolled in School	0.020	Uses Crutches/Walker	0.036			Age	-0.006
Female	0.019	PTSD Diagnosis	0.035			PTSD Diagnosis	0.005
No Regular Exercise	0.018	No Regular Exercise	0.031			No Regular Exercise	0.001
Poor Health	0.018	Has Insurance	0.021				
Household Size	0.008	Age	-0.013			IND	12
Age	-0.007	Social Support	-0.009			INT	1
Social Support	-0.006	Household Size	0.004			HS	2
Age First Intercourse	-0.006	Household Income	-0.002			COM	0
		Hours of Television	0.000				
IND	15						
INT	3	IND	15				
HS	2	INT	3				
COM	0	HS	3				
		COM	0				
PTSD Diagnosis				Anxiety Diagnosis			
Attempt		Ideation		Attempt		Ideation	
Factors	21		12	Factors	16		19
BD	0.687		0.883	BD	0.656		0.871
MCE	0.110		0.164	MCE	0.104		0.161
AUC	0.560		0.576	AUC	0.582		0.585
MSE	0.195		0.272	MSE	0.185		0.267
MAE	0.390		0.545	MAE	0.370		0.535
ACC	0.890		0.836	ACC	0.896		0.839
Intercept	-2.646	Intercept	-1.899	Intercept	-2.816	Intercept	-1.955
Education: < High School	0.550	Received Counseling	0.240	Education: < High School	0.913	Received Counseling	0.247
Received Counseling	0.213	Depression Diagnosis	0.151	Received Counseling	0.197	Education: < High School	0.169
Depression Diagnosis	0.059	Alcohol Consumption	0.091	Parent Education: High School	-0.147	Depression Diagnosis	0.149
Alcohol Consumption	0.053	Not Received Needed Care	0.075	Depression Diagnosis	0.064	Not Received Needed Care	0.123
Parent Education: < High School	0.049	Uses Marijuana	0.068	Uses Marijuana	0.042	Same Sex Romantic Attraction	0.080
Uses Marijuana	0.043	Same Sex Romantic Attraction	0.059	Not Received Needed Care	0.033	Alcohol Consumption	0.079
Not Received Needed Care	0.039	Anxiety Diagnosis	0.052	Poor Health	0.025	Regular Smoking	0.050
Social Support	-0.037	Regular Smoking	0.047	Regular Smoking	0.018	Uses Marijuana	0.047
Anxiety Diagnosis	0.033	No Regular Exercise	0.040	Enrolled in School	0.014	PTSD Diagnosis	0.037
Same Sex Romantic Attraction	0.032	Poor Health	0.031	Age	-0.009	No Regular Exercise	0.036
Has Insurance	0.031	Age	-0.006	Resides in the South	-0.008	Poor Health	0.031
Education: High School	-0.028	Social Support	-0.003	Age First Intercourse	-0.008	Enrolled in School	0.029
Female	0.026			Education: High School	-0.007	Social Support	-0.025
Poor Health	0.016	IND	9	Alcohol Consumption	0.006	Resides in the South	-0.011
Household Size	0.015	INT	1	No Regular Exercise	0.004	Age	-0.008
Black Race	0.008	HS	2	Social Support	-0.002	Household Size	0.008
Age	-0.006	COM	0			Parent Education: High School	-0.004
Outside Redlining District	-0.005			IND	11	Female	0.002
Age First Intercourse	-0.003			INT	2	Hours of Television	0.001
Female	0.002			HS	2		
Body Mass Index	-0.001			COM	1	IND	13
						INT	3
IND	14					HS	2
INT	3					COM	1
HS	3						
COM	1						
Depression Diagnosis				LD Diagnosis			
Attempt		Ideation		Attempt		Ideation	
Factors	16		13	Factors	19		18
BD	0.662		0.900	BD	0.432		0.689
MCE	0.106		0.169	MCE	0.063		0.121
AUC	0.584		0.571	AUC	0.733		0.702
MSE	0.187		0.278	MSE	0.113		0.202
MAE	0.374		0.558	MAE	0.227		0.407
ACC	0.894		0.831	ACC	0.937		0.879
Intercept	-3.038	Intercept	-1.804	Intercept	-3.640	Intercept	-2.745
Education: < High School	1.004	Received Counseling	0.240	Education: < High School	0.914	Received Counseling	0.599
Received Counseling	0.238	Education: < High School	0.172	Received Counseling	0.600	Not Received Needed Care	0.310
Not Received Needed Care	0.090	Not Received Needed Care	0.115	Alcohol Consumption	0.194	Education: < High School	0.291
Uses Marijuana	0.067	Same Sex Romantic Attraction	0.103	Depression Diagnosis	0.143	Depression Diagnosis	0.271
Uses Crutches/Walker	-0.066	Alcohol Consumption	0.096	Female	0.106	Same Sex Romantic Attraction	0.213
Alcohol Consumption	0.039	Regular Smoking	0.073	Regular Smoking	0.094	Alcohol Consumption	0.159
Parent Education: < High School	0.037	Uses Marijuana	0.056	Household Size	0.075	Has Insurance	0.094
Poor Health	0.022	Poor Health	0.031	Not Received Needed Care	0.056	Uses Marijuana	0.092
Regular Smoking	0.012	Enrolled in School	0.026	Poor Health	0.044	Regular Smoking	0.091
Age	-0.009	Age	-0.010	Same Sex Romantic Attraction	0.041	Enrolled in School	0.087
Employed	-0.007	No Regular Exercise	0.004	Social Support	-0.039	Poor Health	0.080
Social Support	-0.006	Employed	-0.003	Received Special Educational Services	0.039	Female	0.080
Enrolled in School	0.006	Household Size	0.002	No Regular Exercise	0.034	PTSD Diagnosis	0.060
Household Size	0.004			Has Insurance	0.019	No Regular Exercise	0.045
Age First Intercourse	-0.002	IND	10	ADHD Diagnosis	-0.012	Social Support	-0.023
Anxiety Diagnosis	0.001	INT	1	Age	-0.012	Age	-0.013
No Regular Exercise	0.003	HS	2	Outside Redlining District	-0.008	ADHD Diagnosis	-0.011
		COM	0	PTSD Diagnosis	0.003	Parent Education: College	0.003
IND	12			Household Income	-0.002		
INT	3					IND	13
HS	2			IND	12	INT	2
COM	0			INT	3	HS	3
				HS	3	COM	0
				COM	1		

*Factors are listed in order of their contribution to the predictive model, with the strongest predictors listed first.

IND, Individual.

INT, Interpersonal.

HS, Health System.

COM, Community.

BD, Binomial Deviance.

MCE, Misclassification Error.

AUC, Area Under the Curve.

MSE, Mean-Squared Error.

MAE, Mean Absolute Error.

ACC, Accuracy.

### Full sample analysis

In the full sample, the LASSO model identified 20 factors (15 individual, 3 interpersonal, 2 health system, 0 community) significantly associated with SA and 21 factors (15 individual, 3 interpersonal, 3 health system, 0 community) for SI. The SA model achieved a Binomial Deviance of 0.610 and a Misclassification Error of 9.5%, with an AUC of 0.601. For SI, the Binomial Deviance was 0.798, the Misclassification Error was 14.1%, and the AUC was 0.600. The five most important factors were individual and health system characteristics, including primarily educational attainment, diagnoses, and health system (receipt of counseling, not receiving needed medical care features.

### ADD/ADHD subgroup

In the ADD/ADHD subgroups, the LASSO model identified 4 factors (3 individual, 0 interpersonal, 1 health system, 0 community) of SA and 15 factors (12 individual, 1 interpersonal, 2 health system, 0 community) of SI. The Binomial Deviance for SA was 0.660, with a Misclassification Error of 10.4% and an AUC of 0.565. For SI, the Binomial Deviance was 0.846, the Misclassification Error was 15.3%, and the AUC was 0.584. The most important factors associated with both SA and SI included depression, receipt of counseling, and educational attainment.

### PTSD subgroup

Among individuals with PTSD, the model identified 21 factors for SA (14 individual, 3 interpersonal, 3 health system, 1 community) and 15 factors for SI (9 individual, 1 interpersonal, 2 health system, 0 community). The performance metrics were slightly better for SA, with a Binomial Deviance of 0.570, Misclassification Error of 10.3%, and AUC of 0.614. The SI model had a Binomial Deviance of 0.787, Misclassification Error of 14.1%, and AUC of 0.589. Those most important factors included both individual and community-level characters. While both receipt of counseling and educational attainment were highly associated among other subgroups, marijuana use, and alcohol consumption had the strongest association among those with PTSD.

### Anxiety subgroup

In the anxiety subgroup, 20 factors (11 individual, 2 interpersonal, 2 health system, 1 community) were associated with SA and 20 for SI (13 individual, 3 interpersonal, 2 health system, 1 community). The SA model’s performance showed a Binomial Deviance of 0.612, Misclassification Error of 9.4%, and AUC of 0.599. The SI model had a Binomial Deviance of 0.756, Misclassification Error of 13.8%, and AUC of 0.592. The most predictive factors included individual and community characteristics. Health-related behaviors including marijuana use, cigarette smoking, and alcohol consumption were highly associated. Moreover, not receiving necessary healthcare and/or rating one’s health as poor was highly associated with STB.

### Depression subgroup

For individuals with Depression, 16 factors (12 individual, 3 interpersonal, 2 health system, 0 community) were identified for SA and 13 (10 individual, 1 interpersonal, 2 health system, 0 community) for SI. The SA model’s performance metrics included a Binomial Deviance of 0.662, Misclassification Error of 10.6%, and AUC of 0.589. For SI, the Binomial Deviance was 0.900, Misclassification Error was 16.9%, and AUC was 0.577. Smoking, alcohol consumption, marijuana use, and educational attainment were, again, the strongest individual level factors associated with both SI and SA, while receipt of counseling and medical care were the strong health system factors.

### LD subgroup

For individuals with LD, 19 factors were identified for SA (12 individual, 3 interpersonal, 3 health system, 1 community) and 18 for SI (13 individual, 2 interpersonal, 3 health system, 0 community). The SA model yielded a Binomial Deviance of 0.432, Misclassification Error of 6.3%, and AUC of 0.689, indicating better performance compared to other subgroups. The SI model had a Binomial Deviance of 0.689, Misclassification Error of 12.1%, and AUC of 0.655. Depression, smoking, and receipt of health care/counseling were the most important factors for both SA and SI, but female sex and household size were much predictive of SA than SI among those with LD.

### Key factors

The LASSO regression models demonstrated varying performance across subgroups, with the LD subgroup showing the highest accuracy with SA. While the models demonstrated only modest predictive accuracy, with an AUC of less than.60 across most subgroups, they performed notably better within the LD subgroup. This suggests that while these models can provide some insight into population-level risk and associated factors potentially requiring intervention, their ability to identify specific individuals at elevated risk is limited, aligning with previous research (30, 31). However, models with modest AUC values can still offer value by identifying broader populations at elevated risk. These models may inform the design of public health programs or policy initiatives by highlighting areas or demographic groups where intervention may be needed. While caution is warranted when using such models at the individual level, they should be used in conjunction with other tools and assessments to ensure more reliable decision-making. The improved performance in the LD subgroup suggests that predictive models may benefit from a more tailored approach when applied to distinct populations. This finding underscores the importance of refining tools and interventions to meet the unique needs of specific subgroups, an approach that can ultimately increase both accuracy and impact.

These results highlight the complex interplay of factors influencing suicide risk and the need for interventions tailored to specific at-risk populations. Across the full sample and subgroups, significant factors for SA and SI included both individual-level (e.g., prior mental health diagnoses, substance use) and interpersonal factors (e.g., family relationships, exposure to violence). Generally, demographic characteristics such as age, sex, and race/ethnicity were not highly associated with STB in any of the models, while substance-related behavior and mental health diagnosis remained persistently impactful. Same sex curiosity and social support were non-negligible contributors to STB among most groups. Health system and community-level factors were less consistently identified across subgroups. The variation in selected factors across subgroups underscores the importance of considering the heterogeneity within populations when assessing risk factors for SI and SA.

## Discussion

The 2024 *National Strategy* emphasizes the need for upstream and comprehensive suicide prevention efforts. To pursue new prevention strategies, research is needed to understand factors associated with STB inclusive of multiple socioecological levels that target SDOH. The Add Health data offered a unique opportunity to examine associations with STB among adolescents followed through mid-life considering multiple individual, interpersonal, health system, and community-level factors. The identified individual-level associations for mental health diagnoses are consistent with prior literature, indicating that individuals who report experiencing SI and/or SA also report high rates of mental health diagnoses including depression and anxiety (3, 9). Notably, those with ADHD or LD and depression were at greater risk for SI/SA, suggesting greater vulnerability for those who are neurodiverse and who may need more support for executive functions such as cognitive flexibility and inhibitory control that could contribute to increased risk for STB. The individual, behavioral factors associated with increased risk, such as excessive television viewing, alcohol intake, smoking, and substance use may be related to experiential avoidance (32). Engaging in these types of avoidance behaviors may distract from experiencing uncomfortable emotions, while also potentially hindering development of self-regulation skills and healthy coping strategies (e.g., regular exercise (33), which was protective in the current study) (34). As elaborated below, individuals experiencing STB frequently reported not receiving needed care; thus, these behaviors may serve as a substitute.

Consistent with prior literature (35, 36), social support was a significant interpersonal-level protective SDOH. A 2022 meta-analysis of social support interventions (37) found pooled evidence that face-to-face social support interventions reduced suicide death, but not reduction in SA. Therefore, despite consistent evidence for the importance of social support, development of effective interventions may still be warranted that address STB type. Increased household size and/or decreased household income were associated SDOH in some models, which may reflect that parents with more children and less financial resources have fewer opportunities to support each individual family member.

Regarding health system related SDOH factors, we consistently found that individuals who received counseling were significantly more likely to report STB, across all models. This likely reflects that individuals who are at-risk for suicidality and/or who are experiencing STB were able to receive at least some type of counseling. However, we cannot determine the type, quality, or duration of counseling nor if it helped to reduce or alleviate their SI or risk for future SA. Notably, “not receiving needed care” was significantly associated across most of the tested models, indicating that additional health services were indicated and unavailable. Interestingly, having insurance—when significant—was not protective, and was in fact associated with increased risk of STB in some models. Merely having insurance may not meet individuals’ needs, either by not covering the specific services they need or because access to the health services needed is not guaranteed even with coverage.

Existing evidence demonstrates that community-level SDOH factors such as geographical socio-economic deprivation are associated with STB (38–41). Living within a redlining district was significantly associated with SA among the PTSD and LD subgroups, suggesting historical geographical discrimination may have a more notable impact on suicide risk for individuals facing other challenges such as LD and history of trauma.

Additional SDOH factors identified as significant at the individual-level may, in fact, be influenced by socioecological factors including policy, cultural beliefs, and access to other service systems (beyond healthcare) that provide support. For example, lower levels of education, lower household income, and sexual minority status were associated with STB in many of the models. A fifteen-year study in the U.S. identified significantly higher rates of suicide death for individuals without a college degree (42), pointing to socioeconomic disparities in suicide risk and echoed in our findings. Further, evidence demonstrates that anti-LGBT legislation negatively impacts mental health and elevates suicide risk among LGBT individuals (43). Additional research could investigate causal links to determine mechanisms through which these individual-level factors may be affected by societal-level influences.

A similar set of factors were identified as significant for SA (15 individual, 3 interpersonal, 2 health system) as well as SI (15 individual, 3 interpersonal, 3 health system). However, there have been emerging findings indicating that distinct sets of risk factors (e.g., individual, psychiatric, psychological) are associated with SI compared to SA (44). Given the differences in clinical severity and prevalence between SI and SA, it is also notable that the LASSO coefficients showed substantial differences in factors considered more important for each outcome, which may indicate distinct priority targets for prevention of SA versus SI.

### Limitations

Although this study provides valuable information on factors associated with STB, the following limitations must be considered. First, only between two and four percent reported SA and seven to 13% reported SI. Small sample sizes have been a consistent concern among ML studies and consequently their generalization (45). Second, all information is self-reported and cannot be validated or verified. Prior research concerning variation in self-reported STB in Add Health showed some variation by race and ethnicity (46). Additionally, studies show that certain health-related behaviors and conditions can suffer from underreporting, delayed reporting, and incomplete reporting (47). Further, survey data can also suffer from recency bias, response bias, recall bias, and favorability bias. Third, not all potential factors associated with STB were available in Add Health. For example, the survey did not contain information on firearm ownership, parental STB, levels of perceived community safety, or mental health policies. Fourth, Add Health employed a complex design and sampling framework that could not be incorporated into the LASSO regression. Fifth, Add Health allowed for examination of multi-level factors associated with STB from adolescence through mid-adulthood, rather than only one time point. Sixth, while LASSO performs both variable selection and regularization to enhance the prediction accuracy and interpretability of the model produced, it has several limitations including variable selection instability, difficulty handling multicollinearity, and limited variable selection in high dimensional data. Seventh, this study presents only a crude measure of SI and does not account for the frequency or severity of suicidal thoughts compared to brief validated psychometric tools. The 12-month timeframe for capturing SI and SA misaligns with the shorter timeframes typically used in formal diagnostic criteria, such as the 2 to 4-week windows for conditions like depression. Longer timeframes increase the risk of recall bias, as individuals may inaccurately remember events from further back in time. Additionally, a 12-month period may obscure temporal trends and fail to capture acute periods of crisis or current suicide risk, which are critical for timely intervention. This inconsistency can also complicate comparisons with studies or assessments using shorter, standardized timeframes, potentially limiting the precision and applicability of findings. Finally, the number of respondents varied in each wave which may have contributed to demographic variation and observation of STB. Additionally, the identified predictors should not be interpreted as causal factors. Further research is needed to establish causal pathways and underlying mechanisms.

## Conclusions

Using a large, nationally representative panel survey, this study applied LASSO regression to identify factors associated with SI and SA among youth and young adults. Results showed that behaviors including alcohol consumption, marijuana use, smoking, no regular exercise, and hours of television viewing were significantly associated with SI and SA for both the full sample as well as most diagnosis subgroups. Increased social support, household size, household income, and parental marital status were significantly related to a lower likelihood of SI and SA. Diagnosis subgroups showed distinct patterns of individual, interpersonal, health system, and community factor associations. These findings can be utilized to identify individuals and subgroups with high-risk of STB and assist public health officials in designing interventions to reduce STB among young adults, particularly those with high-risk diagnoses. Additional research focusing on diagnostic subgroups is needed to identify how co-occurring ADHD, PTSD, depression, anxiety, and LD interact with factors at multiple levels to impact STB.

## Data Availability

The data analyzed in this study is subject to the following licenses/restrictions: Data was acquired through the Add Health Restricted-Use Data Contract #19101801. Requests to access these datasets should be directed to http://www.cpc.unc.edu/projects/addhealth/design.
